# Feasibility of Augmented Reality in Clinical Simulations: Using Google Glass With Manikins

**DOI:** 10.2196/mededu.5159

**Published:** 2016-03-07

**Authors:** Basil Chaballout, Margory Molloy, Jacqueline Vaughn, Raymond Brisson III, Ryan Shaw

**Affiliations:** ^1^Duke UniversityTrinity College of Arts & SciencesDurham, NCUnited States; ^2^Duke UniversitySchool of NursingDurham, NCUnited States

**Keywords:** clinical simulation, augmented reality, feasibility, student learning, Google Glass

## Abstract

**Background:**

Studies show that students who use fidelity-based simulation technology perform better and have higher retention rates than peers who learn in traditional paper-based training. Augmented reality is increasingly being used as a teaching and learning tool in a continual effort to make simulations more realistic for students.

**Objective:**

The aim of this project was to assess the feasibility and acceptability of using augmented reality via Google Glass during clinical simulation scenarios for training health science students.

**Methods:**

Students performed a clinical simulation while watching a video through Google Glass of a patient actor simulating respiratory distress. Following participation in the scenarios students completed two surveys and were questioned if they would recommend continued use of this technology in clinical simulation experiences.

**Results:**

We were able to have students watch a video in their field of vision of a patient who mimicked the simulated manikin. Students were overall positive about the implications for being able to view a patient during the simulations, and most students recommended using the technology in the future. Overall, students reported perceived realism with augmented reality using Google Glass. However, there were technical and usability challenges with the device.

**Conclusions:**

As newer portable and consumer-focused technologies become available, augmented reality is increasingly being used as a teaching and learning tool to make clinical simulations more realistic for health science students. We found Google Glass feasible and acceptable as a tool for augmented reality in clinical simulations.

## Introduction

Patient simulation is a useful tool for training students and ascertaining competency prior to students entering clinical environments [1]. Simulations using patient manikins allow students to acquire necessary skills and practice without fear of harming patients. In order for knowledge gained during patient simulations to translate into clinical practice, scenarios must feel realistic to students.

Studies show that students who use fidelity-based simulation technology perform better and have higher retention rates than peers who learn in traditional paper-based training [2]. Using medium- and high-fidelity simulation manikins is an effective teaching and learning method for health science education [3]. Developing care delivery skills in a simulation practice setting enables students to focus on performance, which can enhance patient safety [4].

Augmented reality, which combines virtual reality with physical materials, instruments, and feedback, is increasingly being used as a teaching and learning tool to make simulations even more realistic for students. Studies show that this level of realism is good for medical training and results in significantly improved skills transfer in students [5,6].

The aim of this feasibility and acceptability trial was to assess the use of Google Glass as a tool to enhance the realism of high-fidelity simulations for training health science students.

##  Methods

### Development

Students watched a video while wearing Google Glass (see Multimedia Appendix 1) of a patient actor simulating respiratory distress. Google Glass is a head-mounted device with a built-in camera, display, touchpad, battery, and microphone worn like a set of spectacles. It allows data to be free from desktop or laptop computers and portable devices and places a nonobstructive video image in the upper right-hand corner of the user’s field of vision.

In this project, Google Glass allowed for a video to appear in each student’s field of view without the need to look at a separate video screen while delivering care to a manikin. The video was that of a standardized patient portraying a patient experiencing respiratory distress.

An actor who strongly resembles the manikin acted out the specific scenario the students would be responding to in the simulation. The video presented a car accident victim who was having difficulty breathing while simultaneously panicking. It was filmed with high definition cameras outside the use of Google Glass and shot as a constant stream to be played entirely from start to finish during the simulation. It was produced to match the expected duration and procedure of the simulation to reflect what the students were performing on the manikin. This video was placed on the video-sharing website YouTube and was viewed through the Glass prism during the simulation experience.

After the trial received approval from the university’s institutional review board, student volunteers were recruited to participate in the scenarios while wearing Google Glass. The video of the patient was played in each student’s field of vision to augment the clinical changes the manikin was programed to display. The intent was for the patient in the video to show in real time what the manikin was displaying. Instructors sat behind a one-way mirror in a control room to view the scenario and witness what the participants were viewing by watching the video at the same time on a computer. A debriefing followed the simulation experience. During the debriefing, students were given the opportunity to reflect on the simulation experience.

### Measures

####  Feasibility

Feasibility was assessed as our ability to set up Google Glass, play a video of the patient actor in the student’s field of vision during a simulation, and overcome technical challenges.

#### Acceptability

Following the scenarios, participants were asked if they would recommend continued use of this technology in clinical simulation experiences. In addition, they completed two surveys and answered an open-ended question about their experience. The 13-item Student Satisfaction and Self-Confidence in Learning Scale was designed to measure these outcomes using a 5-point scale from 1 (strongly disagree) to 5 (strongly agree). Reliability was previously reported as a Cronbach alpha of .94 to .92 for the satisfaction subscale and .87 to .83 for the self-confidence subscale [7,8]. The 20-item Simulation Design Scale evaluates objectives and information, support, problem solving, feedback, and fidelity of a clinical simulation. Reliability was previously reported as a Cronbach alpha of .96 for the overall scale [8].

### Statistical Analysis

Descriptive statistics were calculated from the survey data and analyzed using SPSS version 23.0 (IBM Corp). Qualitative data from the open-ended question were interpreted using content analysis.

##  Results

###  Feasibility

The quality of the video was realistic. We were able to upload the video to YouTube without difficulties, and students were able to watch it in Google Glass during the simulations.

There were a few challenges. Due to security measures put in place by our hospital, it was difficult to connect the device to a wireless network in order to view the video on YouTube. The students faced a large learning curve when attempting to use the device for the first time, and it was hard to coordinate the video to start as soon as the actual simulation began. Google Glass had a short battery life and a tendency to overheat when used for a long duration. Nevertheless, the video played during all simulations.

### Acceptability

Most students recommended we continue using the Google Glass technology in clinical simulations (10/12, 80%). The remaining students reported they were unsure about continued use of the technology. Students reported high mean scores on the design of the simulation and satisfaction with the simulation to promote learning, and self-confidence in learning. Table 1 shows the results from the Simulation Design Scale and Student Satisfaction and Self-Confidence in Learning surveys.

**Table 1:** Acceptability of Google Glass in a clinical simulation.

Measures		Mean (SD)
**Simulation Design Scale**		
	Objectives and information	4.65 (0.18)
	Support	4.85 (0.04)
	Problem solving	4.53 (0.30)
	Feedback/guided reflection	4.85 (0.14)
	Fidelity (realism)	4.67 (0.12)
**Student Satisfaction and Self-Confidence in Learning**		
	Satisfaction with current learning	4.67 (0.13)
	Self-confidence in learning	4.35 (0.60)

The mix of opinions, however, was more directly seen in the answers to the open-ended questions. One student reported, “It was kind of distracting. I think I might just have to get used to [it]. The reason why is because my eyes have to look at two things.” Another student indicated that the video actually helped him understand the respiratory distress of the patient by stating, “After it [the video] got going it just became part of the simulation.” Students expressed a desire to have more information presented in Google Glass and asked for the ability to incorporate live vital signs into the display. Students also wanted prior familiarization with the technology and preparation for technological difficulties.

## Discussion

Google Glass has been used in clinical simulation-based training for capturing video during care delivery [9,10] and ultrasound-guided venous access [11] and as a tool for pediatric surgery [9]. This study is the first to our knowledge to use it as a tool to mimic a manikin by displaying the video in the user’s field of vision. We found that it was feasible to use Google Glass as an augmented reality tool for learning in clinical simulations. Students successfully watched a video in their field of vision of a patient that mimicked the simulated manikin they were caring for. The purpose of augmenting reality during the simulation instead of playing the video prior to it was to give a better perception of working with an actual patient in real time. Students spoke of the benefits of being able to view a patient during the simulation, reported perceived realism with the technology, and recommended using the technology in the future.

While it was feasible to use the technology in the simulations, there were challenges. We encountered technical barriers when setting up the devices, and it took time to train students on how to use them. The learning curve may have impacted student perceptions of the usefulness of the technology. For example, one student experienced a delay between the Google Glass video and the live simulation due to technical difficulties and confusion on how to work the device. Attention may have been compromised and problem-solving skills momentarily reduced because students were learning how to use the device while using the device during a simulated clinical experience.

### Limitations

Because this was a small sample and our main goal was to discover the feasibility and acceptability of using this technology in a clinical simulation, we cannot make conclusions as to the benefit of this technology on learning outcomes. Nevertheless, the similarity in data and the increased perception of realism point to this being a promising proof of concept worthy of future testing.

### Conclusions

As newer portable and consumer-focused technologies become available, augmented reality is increasingly being used as a teaching and learning tool to make clinical simulations more realistic for health science students [11,12,13]. We found Google Glass feasible and acceptable as a tool for augmented reality in clinical simulations.

## Multimedia Appendix 1

Duke Google Glass for Clinical Simulations.
